# Short-Term Overfeeding Increases Circulating Adiponectin Independent of Obesity Status

**DOI:** 10.1371/journal.pone.0074215

**Published:** 2013-08-30

**Authors:** Farrell Cahill, Peyvand Amini, Danny Wadden, Sammy Khalili, Edward Randell, Sudesh Vasdev, Wayne Gulliver, Guang Sun

**Affiliations:** 1 Division of Medicine, Faculty of Medicine, Memorial University of Newfoundland, St. John’s, NL, Canada; 2 Discipline of Laboratory Medicine, Memorial University of Newfoundland, St. John’s, NL, Canada; University of Cordoba, Spain

## Abstract

**Background:**

Adiponectin is an adipose tissue derived hormone which strengthens insulin sensitivity. However, there is little data available regarding the influence of a positive energy challenge (PEC) on circulating adiponectin and the role of obesity status on this response.

**Objective:**

The purpose of this study was to investigate how circulating adiponectin will respond to a short-term PEC and whether or not this response will differ among normal-weight(NW), overweight(OW) and obese(OB).

**Design:**

We examined adiponectin among 64 young men (19-29 yr) before and after a 7-day overfeeding (70% above normal energy requirements). The relationship between adiponectin and obesity related phenotypes including; weight, percent body fat (%BF), percent trunk fat (%TF), percent android fat (%AF), body mass index (BMI), total cholesterol, HDLc, LDLc, glucose, insulin, homeostatic model assessment insulin resistance (HOMA-IR) and β-cell function (HOMA-β) were analyzed before and after overfeeding.

**Results:**

Analysis of variance (ANOVA) and partial correlations were used to compute the effect of overfeeding on adiponectin and its association with adiposity measurements, respectively. Circulating Adiponectin levels significantly increased after the 7-day overfeeding in all three adiposity groups. Moreover, adiponectin at baseline was not significantly different among NW, OW and OB subjects defined by either %BF or BMI. Baseline adiponectin was negatively correlated with weight and BMI for the entire cohort and %TF, glucose, insulin and HOMA-IR in OB. However, after controlling for insulin resistance the correlation of adiponectin with weight, BMI and %TF were nullified.

**Conclusion:**

Our study provides evidence that the protective response of adiponectin is preserved during a PEC regardless of adiposity. Baseline adiponectin level is not directly associated with obesity status and weight gain in response to short-term overfeeding. However, the significant increase of adiponectin in response to overfeeding indicates the physiological potential for adiponectin to attenuate insulin resistance during the development of obesity.

## Introduction

The accumulation of white adipose tissue (WAT) is the product of a chronic positive energy balance in an obesogenic environment [1]. Numerous disorders such as insulin resistance, type 2 diabetes (T2D) and cardiovascular disease have shown a strong association with the accumulation of subcutaneous and visceral fat mass [2], [3]. Once regarded as a site for energy storage, recent research has shown that WAT secretes a large number of physiologically active proteins [4], [5], [6] thus playing an integral role in human endocrinology and energy homeostasis [7], [8], [9]. Adiponectin, for example, is predominantly secreted into circulation from adipose tissue, which increases insulin sensitivity resulting in the effective disposal of glucose from circulation [10], [11], [12]. Accumulating evidence suggests that adiponectin is strongly associated with glucose and lipid metabolism, although adiponectin’s role in the development of obesity and insulin resistance remains unclear.

Considering that adiponectin has been recognized as a significant insulin sensitizer it would be consistent to anticipate that circulating adiponectin levels would be considerable in the presence of an insulin resistant state such as obesity. However, the puzzling actuality suggests that adiponectin is inversely associated with obesity [13], [14], [15], [16], [17], [18] and insulin resistance [16], [19], [20], [21]. Consequently if adiponectin is an anti-obesigenic and anti-diabetic protein, expected to increase in circulation, it is perplexing that adiponectin levels are attenuated in both obese and diabetic populations. Nevertheless there is significant evidence that adiponectin does indeed play a beneficial role in both lipid and glucose metabolism [20], [21], [22], [23]. Rodent studies have shown that adiponectin gene knockout mice severely attenuate glucose disposal and [20], [23] the administration of adiponectin can effectively ameliorate insulin resistance [21], [22]. These findings demonstrate that adiponectin can protect or even reverse obesity related disease states. However, the paradoxical inverse relationship of adiponectin with adiposity remains unclear.

Our laboratory and others have demonstrated that a short-term positive energy challenge affects glucose and lipid metabolism, which results in significant body weight gain and insulin resistance [1], [24], [25], [26], [27]. Moreover, changes in nutritional status have also shown to significantly affect circulating adipokines [1], [24], [27]. For this reason we propose that investigating the response of adiponectin, to adiposity development, will provide insight into the potential role that adiponectin may play in the development of obesity and diabetes. The objective of our present investigation is to address the following as yet unanswered question: Does circulating adiponectin increase in response to a short-term positive energy challenge and is this potential response obesity status dependent. To our knowledge this is the first study of its kind to investigate the effect of a 7-day positive challenge (70% above normal energy requirements) on circulating adiponectin among normal-weight, overweight and obese individuals.

## Methods

### Ethics Statement

This study was approved by The Health Research Ethics Authority (HREA) for the Faculty of Medicine, Memorial University of Newfoundland and Labrador, St John’s, Canada. All subjects provided written informed consent. Initial data collection for this study began October 2003.

### Subjects

Sixty-four young men, recruited from the city of St. John’s (Newfoundland and Labrador, Canada) and surrounding areas, participated in the current positive energy challenge (PEC) study. The study inclusion criteria were as follows: 1) male; 2) non-smoker, 3) 19–29 yr of age; 4) at least third-generation Newfoundlander; 5) healthy young men, without any serious metabolic, cardiovascular, or endocrine disease; 6) not receiving medication for lipid metabolism; 7) reported stable weight (±2.5 kg) in the previous 6 months and 8) subjects abstained from any alcoholic or additional calorie-containing beverage consumption during the study period. Participants refrained from taking any drugs/medications throughout the duration of the study. All physical and biochemical measurements were collected, after a 12 hour fast, on the morning of the first day of overfeeding and one day after the 7^th^ and final day of overfeeding. Study participants were free living throughout the experiment and were not restricted to an experimental environment, such as a hospital.

### Serum measurements

Fasting blood samples were obtained from all subjects before and after the completion of the overfeeding intervention. Serum was stored at –80°C for subsequent analyses. The majority of biochemical markers [28] and adiponectin [29] remain stable under these storing conditions. Adiponectin (Phoenix Pharmaceuticals, Belmont, California) concentrations were measured in duplicate utilizing enzyme-linked immunosorbent assays. The intra-assay variation ranged from 4.8 – 5.4% and the inter-assay variation was 5.1%. The detection limit of the adiponectin ELISA kits used was 0. 15 ng/ml when using 100 µL diluted samples. Serum insulin concentrations were measured with the use of an immunoassay analyzer (Immulite; DPC, Los Angeles, CA). The homeostasis model assessment (HOMA) was used as a measure of insulin resistance and beta cell function [30]. 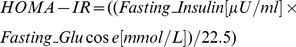


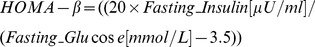



Serum concentrations of glucose, triacylglycerols (TG), high-density lipoprotein (HDLc) cholesterol and total cholesterol were measured by an Lx20 analyzer (Beckman Coulter Inc, Fullerton, CA). Low-density lipoprotein (LDLc) cholesterol was calculated as seen below. The LDL cholesterol calculation is reliable in the absence of severe hyperlipidemia.
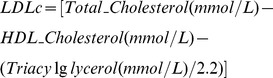



### Body composition measurements

Height (nearest 0.1 cm) and weight (nearest 0.1 kg) measurements were collected and Body Mass Index (BMI) calculated. BMI was defined as weight divided by height squared (kg/m^2^). Percent body fat (%BF), percent trunk fat (%TF), and percent android (abdominal) fat (%AF), were measured utilizing dual-energy X-ray absorptiometry (DXA, Lunar Prodigy; GE Medical Systems, Madison, WI). Measurements were performed on subjects in a supine position, after the removal of all metal accessories [1], [31]. Version 12.2 of the enCORE (enCORE,Ver 12.2, 2008, GE Medical Systems, Madison, WI) software package was used for DXA analysis. DXA measurements (fat weight, lean tissue weight and bone weight) are recorded to the nearest 0.001 kg.

### Overfeeding protocol

Participants consumed 70% more calories than their normal energy requirements (hypercaloric), which consisted of 15% protein, 35% fat, and 50% carbohydrates to mimic the common daily diet in North America. Baseline energy requirements were determined from three 24-hr diet recalls and a 30-d dietary inventory. The average caloric intake per day was as follows: energy (baseline: 2969 kcal; overfeeding: 5471 kcal), protein (baseline: 106 g; overfeeding: 178 g), carbohydrates (baseline: 394 g; overfeeding: 713 g), fiber (baseline: 19 g; overfeeding: 33 g), total fat (baseline: 105 g; overfeeding: 221 g), saturated fat (baseline: 38 g; overfeeding: 71 g), and cholesterol (baseline: 304 mg; overfeeding: 735 mg). Subjects were offered meals at 0900, 1200, and 1700h every day, and energy values and macronutrient content of the food were measured by using FOOD PROCESSOR SQL software (version 9.5.0.0; ESHA Research, Salem, OR). The actical physical activity monitor (Mini Mitter Co, Inc, Bend, OR) was used to determine the total energy expenditure. Physical activity levels were controlled below 15% between the baseline and overfeeding period. Full details of our overfeeding protocol have been previously described and all of the participants in this study were members of these studies [1], [24], [25]. On average, subjects gained 2.2±1.4 kg body weight, of which 44.1±30.4% (0.842 kg±0.531) was body fat. A 7-day overfeeding protocol, 70% above normal energy requirements, was chosen for this study to ensure that the intervention would induce metabolic changes. [1], [24], [25].

### Statistical analysis

Data are presented as means ± SDs unless otherwise stated. Before any statistical analysis was performed, data that were not normally distributed were logarithmically transformed (TG, insulin, HOMA-IR and HOMA-β) to approximate normal distribution. Sixty-four young men were used for the analysis of this paper. Subjects were classified on the basis of %BF as normal weight (8 – 20.9%), overweight (21 – 25.9%) or obese (> 26%) according to criteria recommended by Bray [32]. Statistical analysis was also performed on adiposity groups classified by BMI according to the WHO criteria [33]. To further explore the relationship between adiponectin and body composition, participants were divided into tertiles according to baseline fasting serum adiponectin concentrations (µg/ml) as follows: Low (Bottom 33.3%), Medium (middle 33.3%) and High (top 33.3%) adiponectin concentration. The range of serum adiponectin at baseline for the Low, Medium and High adiponectin concentration groups were 1.60–8.15 µg/ml, 8.20–13.80 µg/ml, and14.20–27.10 µg/ml respectively.

Differences in variables between the three adiposity groups in response to overfeeding were analyzed by using a mixed model repeated two-way analysis of variance. Baseline values between the three adiposity groups were analyzed using a one-way analysis of variance. The Bonferroni post-hoc test was run after the one-way ANOVA and the two-way ANOVA displaying a significant overfeeding by adiposity interaction. Within group analysis on the response to overfeeding was performed with a student paired t-test on variables that displayed a significant overfeeding by adiposity interaction effect. Pearson’s correlation analyses were performed to screen for potential factors related to fasting adiponectin concentrations followed by partial correlation analyses after controlling for age. Bonferroni testing was applied to correct for multiple comparisons. Two correlation analyses were performed as follows: 1) adiponectin at baseline was compared with all variables at baseline; and 2) adiponectin at baseline was compared with changes in all variables in response to overfeeding to investigate whether baseline adiponectin could predict the changes in related markers. SPSS software version 18.0 (SPSS Inc, Chicago, IL, USA) was used for all analyses. Statistical analyses were 2-sided and p<0.05 was considered to be statistically significant.

## Results

### Comparison of characteristics at baseline and in response to short-term overfeeding

Physical and biochemical characteristics of subjects at baseline are shown in **Table 1**. Fasting serum adiponectin concentrations at baseline for normal weight (11.60±6.3 µg/ml), overweight (12.84±4.6 µg/ml) and obese (10.69±6.3 µg/ml) subjects were not significantly different from one another. Adiponectin concentrations were also analyzed according to BMI criteria where no significant differences were found (data not shown). 

**Table 1:** Physical and biochemical characteristics of subjects at baseline and in response to 7-days of overfeeding^1^.

	Normal Weight^2^						Overweight^2^						Obese^2^					
	(n = 23 – 25)						(n = 13 – 14)						(n = 22 – 25)					
	Before			After			Before			After			Before			After		
Age (y)	23.9	±	3.7	NA			22.0	±	3.1	NA			23.2	±	2.4	NA		
Height (cm)	178.9	±	6.6	NA			179.6	±	4.8	NA			179.6	±	7.1	NA		
Weight (kg) 4,5,6	72.3	±	9.6	74.4	±	9.6	77.8	±	4.3	79.4	±	4.3	91.4	±	15.4	93.9	±	16.0
BMI (kg/m^2^) 4,5,6	22.6	±	2.7	23.2	±	2.9	24.1	±	1.3	24.6	±	1.5	28.2	±	4.2	29.1	±	4.3
Percent Body Fat (%) 3,5,6,7	14.82	±	3.4	15.60	±	3.4 8	22.54	±	0.8	22.82	±	1.1 8	31.15	±	4.9	31.01	±	4.8
Percent Trunk Fat (%) 3,5,6,7	16.78	±	3.7	17.78	±	3.8 8	25.39	±	1.9	25.79	±	2.2 8	35.07	±	5.4	34.97	±	5.3
Percent Android Fat (%) 3,5,6	19.38	±	4.4	20.16	±	5.0	28.84	±	2.6	29.45	±	2.7	40.00	±	7.2	40.77	±	6.8
Total Cholesterol (mmol/L) 5	4.41	±	0.9	4.68	±	0.9	4.63	±	0.9	4.72	±	1.1	4.59	±	0.7	4.86	±	0.8
HDL-Cholesterol (mmol/L) 5	1.38	±	0.3	1.48	±	0.3	1.38	±	0.3	1.43	±	0.3	1.12	±	0.2	1.34	±	0.3
LDL-Cholesterol (mmol/L)	2.61	±	0.7	2.64	±	0.7	2.82	±	0.7	2.83	±	0.9	2.81	±	0.7	2.83	±	0.6
Triacylglycerol (mmol/L) 5,6	0.94	±	0.3	1.22	±	0.8	0.92	±	0.3	1.00	±	0.5	1.35	±	0.7	1.57	±	0.9
Glucose (mmol/L)	4.98	±	0.4	5.03	±	0.5	5.03	±	0.4	5.09	±	0.6	5.24	±	0.7	5.11	±	0.5
Insulin (pmol/L) 4,5,6	44.22	±	23.8	64.02	±	23.7	51.54	±	17.03	68.69	±	43.6	80.19	±	53.3	108.2	±	76.7
HOMA-IR 4,5,6	1.43	±	0.8	2.09	±	0.9	2.35	±	2.68	2.95	±	2.9	2.80	±	2.3	3.67	±	2.8
HOMA-β 4,5,6	85.45	±	39.38	126.3	±	49.9	101.49	±	25.1	140.8	±	101.7	134.95	±	63.0	190.3	±	107.3
Adiponectin (µg/mL) 5	11.6	±	6.3	13.96	±	4.5	12.84	±	4.6	14.81	±	4.05	10.69	±	6.3	12.82	±	6.1

1 All values are means ± SDs. HOMA - IR, Homeostasis model assessment of insulin resistance; HOMA - β of β cell function; NA, not applicable.

Adiposity status and response to overfeeding analyzed by 2 - factor mix model ANOVA (SPSS, version 17.0 Chicago, IL, USA) for repeated measures.

2 Subjects were classified on the basis of %BF as either normal weight (8 – 20.9%), overweight (21 – 25.9%) or obese (> 26%) according to criteria recommended by Bray [32].

3 Significant difference between normal weight, overweight and obese subjects at baseline (1 - Way ANOVA, followed by a Bonferroni corrected test, P<0.05).

4 Significant difference between normal weight vs obese subjects at baseline (1 - Way ANOVA, followed by a Bonferroni corrected test, P<0.05).

5 Significant difference due to overfeeding (2 - Way mixed model ANOVA, P<0.05).

6 Significant difference due to adiposity status (2 - Way mixed model ANOVA, P<0.05).

7 Significant overfeeding by adiposity status interaction (2 - Way mixed model ANOVA, followed by a Bonferroni corrected test when significant, P<0.05).

8 Significant difference within group (paired t-test, P<0.05).

Changes in body composition and phenotypes of glucose metabolism and lipids in response to the 7-day overfeeding are also described in **Table 1**. The 7-day positive energy challenge protocol significantly increased subcutaneous body fat, serum lipids (except LDL cholesterol), insulin resistance and β cell function. The only significant overfeeding x adiposity status interactions found from our analysis were for %BF and %TF, indicating that normal weight subjects experienced a greater significant increase in body fat percentage than overweight and obese (**Table 1**). The analysis of variance revealed that adiponectin concentrations were significantly increased due to the 7-day positive energy challenge (p<0.0001). Fasting adiponectin levels increased by 18.9% within the entire cohort after overfeeding while increasing by 20.3%, 15.4% and 19.9% among normal-weight, overweight and obese young men respectively. With a ∼19% change in adiponectin due to overfeeding with 64 subjects the calculated power was 0.81.

### Correlations of adiponectin with phenotypes of glucose and lipid metabolism

Correlations between baseline adiponectin and baseline phenotypes were assessed (**Table 2**). In the entire cohort at baseline, adiponectin was negatively correlated with weight and BMI along with a positive correlation with HDLc. However, when these analyses were repeated according to adiposity status no correlation between adiponectin and these variables were found. In addition, when insulin and HOMA-IR were controlled for during partial correlation analysis on the entire cohort the relationship between adiponectin with weight and BMI were nullified. Adiponectin was significantly inversely correlated with %TF, glucose, insulin, and HOMA-IR in the obese group. Correlations between baseline adiponectin and the changes in phenotypes were also assessed to determine if adiponectin could act as a predictor of these parameters under conditions of an energy surplus (**Table 3**). Only two significant negative correlations between baseline adiponectin and the change in phenotypes due to overfeeding were found among the adiposity groups. The first inverse relationship was found between adiponectin and insulin in the normal weight group and the second inverse relationship was found between adiponectin and triacyglycerol in the obese group.

**Table 2:** Partial correlations of baseline variables related to baseline fasting serum adiponectin, after control for age^1^.

	All Subjects		Normal weight^2^		Overweight^2^		Obese^2^	
	(n = 59 – 64)		(n = 23 – 25)		(n = 14)		(n = 22 – 25)	
	r	p	r	p	r	p	r	p
Weight	–0.246	0.050^3^	–0.284	NS	–0.425	NS	–0.177	NS
BMI	–0.289	0.023^3^	–0.322	NS	–0.188	NS	–0.296	NS
Percent Body Fat	–0.127	NS	–0.094	NS	0.338	NS	–0.309	NS
Percent Trunk Fat	–0.155	NS	–0.096	NS	0.235	NS	–0.403	0.041^3^
Percent Android Fat	–0.140	NS	0.064	NS	0.066	NS	–0.343	NS
Total Cholesterol	0.057	NS	0.340	NS	0.050	NS	–0.313	NS
HDL-Cholesterol	0.401	0.001	0.178	NS	0.222	NS	0.357	NS
LDL-Cholesterol	0.020	NS	0.328	NS	–0.039	NS	–0.242	NS
Triacylglycerols	–0.175	NS	0.089	NS	0.167	NS	–0.339	NS
Glucose	–0.225	NS	0.196	NS	0.026	NS	–0.427	0.042^3^
Insulin	–0.225	NS	0.126	NS	–0.192	NS	–0.499	0.015^3^
HOMA-IR	–0.229	NS	0.141	NS	–0.170	NS	–0.505	0.014^3^
HOMA-Beta	–0.204	NS	0.042	NS	–0.292	NS	–0.399	NS

1 HOMA-IR, Homeostasis model assessment of insulin resistance; HOMA-β of β cell function. Partial correlation analysis after control for age was used to screen for the potential changes in factors due to overfeeding related to fasting adiponectin.

2 Subjects were classified on the basis of %BF as either normal weight (8–20.9%), overweight (21–25.9%) or obese (>26%) according to the Bray criteria [32].

3 Not significant after Bonferroni correction to adjust for the multiple variables tested.

**Table 3:** Partial correlations of changes in variables due to overfeeding related to baseline fasting serum adiponectin, after control for age^1^.

	All Subjects		Normal weight^2^		Overweight^2^		Obese^2^	
	(n = 59 – 64)		(n = 23 – 25)		(n = 14)		(n = 22 – 25)	
	r	p	r	p	r	p	r	p
Weight	–0.043	NS	0.015	NS	–0.103	NS	–0.049	NS
BMI	–0.045	NS	0.011	NS	–0.103	NS	–0.050	NS
Percent Body Fat	0.066	NS	0.155	NS	–0.415	NS	–0.027	NS
Percent Trunk Fat	0.101	NS	0.158	NS	–0.248	NS	0.183	NS
Percent Android Fat	–0.123	NS	–0.191	NS	–0.244	NS	–0.057	NS
Total Cholesterol	0.053	NS	0.146	NS	0.326	NS	–0.069	NS
HDL-Cholesterol	–0.086	NS	0.05	NS	–0.073	NS	–0.115	NS
LDL-Cholesterol	0.168	NS	0.241	NS	0.439	NS	–0.040	NS
Triacylglycerols	–0.142	NS	–0.035	NS	–0.430	NS	–0.435	0.038^3^
Glucose	–0.045	NS	–0.264	NS	0.095	NS	0.002	NS
Insulin	–0.125	NS	–0.438	0.032^3^	0.313	NS	0.112	NS
HOMA-IR	–0.084	NS	–0.175	NS	0.059	NS	0.230	NS
HOMA-Beta	–0.081	NS	–0.298	NS	0.210	NS	0.099	NS

1 HOMA-IR, Homeostasis model assessment of insulin resistance; HOMA-β of β cell function. Partial correlation analysis after control for age was used to screen for the potential changes in factors due to overfeeding related to fasting adiponectin.

2 Subjects were classified on the basis of %BF as either normal weight (8–20.9%), overweight (21–25.9%) or obese (>26%) according to the Bray criteria [32].

3 Not significant after Bonferroni correction to adjust for the multiple variables tested.

### Comparison of body composition and lipid profile measures in low, medium and high baseline fasting serum adiponectin concentrations

Lastly, we wanted to investigate the relationship between low, medium, and high baseline fasting serum adiponectin concentrations and markers for lipids and body composition. The between group comparison at baseline demonstrated that there were no significant differences in body composition measurements among the low, medium and high adiponectin concentration groups. Total %BF stratified in the low, medium, and high adiponectin concentrations were 23.9±9.4%, 21.9±8.4% and 22.85±6.8% respectively (p = 0.735). BMI values at baseline were also not significantly different among the low, medium, and high adiponectin concentrations groups. It would seem that only HDL cholesterol was significantly different between the low and medium (p = 0.044) as well as the low and high (p = 0.01) baseline adiponectin concentration groups. The two-way ANOVA revealed that the 7-day overfeeding effectively increased whole adiponectin concentrations (p<0.0001) and in addition that there was a significant overfeeding by adiponectin concentration group interaction (p = 0.002). Sub-group analysis revealed that adiponectin significantly increased within the low baseline adiponectin group from 5.39±2.0 µg/ml to 9.11±3.75 µg/ml (p<0.001) and the medium baseline adiponectin group from 10.61±1.7 µg/ml to 13.05±3.1 µg/ml (p<0.001), but did not significantly increase within the high baseline adiponectin group (18.24±3.6 µg/ml to 18.71±2.8 µg/ml).

## Discussion

The noteworthy finding of the present investigation is that circulating adiponectin levels are significantly increased by a short-term positive energy challenge independent of adiposity status. Taking into consideration that the majority of human cross-sectional studies show that circulating adiponectin is diminished among the obese population [13], [14], [15], [16], and significantly promoted after weight reduction [15], [19], [34], [35], the increase in circulating adiponectin we observed after weight gain in overweight and obese subjects was unexpected. White adipose tissue being the primary location for the adiponectin production, together with the paradoxical inverse relationship between adiponectin and obesity, advocates that circulating adiponectin may be regulated by an adiposity feedback inhibition [15]. Rodent studies, observing a suppression of adiponectin in obese and ob/ob mice [21], [36], [37], support the notion of an adiposity regulated feedback mechanism. However adiponectin transgenic ob/ob mice effectively ameliorating insulin resistance but not obesity [38], [39] and adiponectin knockout mice developing insulin resistance without affecting weight or adiposity [20], [23], provides the strongest evidence that adiponectin is not mediated by adiposity alone. In addition, the administration of adiponectin to ob/ob, db/db, and adiponectin knockout mice can ameliorate the development of insulin resistance without significantly influencing adiposity [21], [22]. In the present study adiponectin concentration significantly increased within the entire cohort and the normal-weight, overweight, and obese subgroups due to overfeeding. However, there was no significant difference in the increase of circulating adiponectin between the three obesity groups due to overfeeding. To further explore this unique finding we stratified our subjects into a low, medium and high adiponectin concentration groups to determine whether adiponectin concentration was associated with the increase in adiposity due to the positive energy challenge. Consequently, there was no significant difference in the increase of our adiposity measurements among adiponectin concentration groups before or after overfeeding. These findings would suggest that there is a definite capacity, regardless of obesity status, for the human body to significantly synthesize and secrete more adiponectin into circulation after being exposed to a short-term overfeeding. To our knowledge this is the first study of its kind to demonstrate that adiponectin levels will significantly increase after a short-term positive energy challenge independent of obesity status.

Our laboratory has demonstrated that a short-term positive energy challenge can significantly affect both physical and biochemical obesity-related phenotypes [1], [24], [25]. However, to date, very few other overfeeding studies have been performed due to the inherent difficulty to carry out this type of study. One 5-day high-fat (60% fat, 32.5% carbohydrates and 7.5% protein) overfeeding (50% greater than normal caloric intake) investigation on 26 normal-weight young men observed a significant increase in adiponectin concentration [27]. Although the age and body weight of these subjects were comparable to the normal-weight group from our current study, the overfeeding intervention and diet composition were considerable different. This study also did not contain any overweight and/or obese subjects. Nevertheless our findings, with those of Bron et al. [27], provides evidence that the significant increase of adiponectin due to overfeeding is likely driven by an increase in total caloric intake rather than macronutrient composition. A 4-week positive energy challenge on 18 young normal-weight adults (12 male and 6 female) did not induce a significant increase in circulating adiponectin [40]. Although no explanation was provided, low statistical power due to the small number of subjects could be a potential reason. Finally, a long-term (100-day) overfeeding study investigated the influence of chronic food intake on circulating adiponectin in 12 pairs of young male identical twins [41]. Study participants were overfed by 840 kcal for 100 days and adiponectin concentration was found to have decreased. However there was no relationship between baseline adiponectin and adiposity before or after prolonged overfeeding. A replication study would be favored since this is the only long-term positive energy challenge study containing a fairly small sample size. Overall, the current human overfeeding studies demonstrate that adiponectin concentrations are up regulated during a short-term positive energy challenge. Our findings would suggest that adiponectin plays a protective role during obesity development and its role is more potent during the early stages of the chronic positive energy balance. This assumption is supported by data from an animal experiment which found that circulating adiponectin concentrations significantly increased in C57BL/6J mice on a high-fat diet during the initial stages of adiposity development [42] and decreased after the full 18 week diet.

The present investigation did not find a significant difference in baseline adiponectin concentration among normal-weight, overweight and obese subjects nor were any associations found between adiponectin concentration and adiposity measurements for these groups. In addition, no significant difference was observed for any body composition measurement among the low, medium and high adiponectin concentration groups. The data from this investigation does not support the hypothesis that adiposity status is a strong contributing factor to circulating adiponectin concentration. We did observe that circulating adiponectin was inversely correlated with BMI and weight for the entire cohort along with %TF in obese subjects. However, recent studies have advocated that the inverse relationship between adiponectin and adiposity phenotypes is solely dependent upon the development of insulin resistance [10], [13], [17], [43]. Therefore, after controlling for fasting insulin and/or HOMA-IR in our analysis the significant partial correlation between adiponectin and aforementioned body composition measurements were nullified. Therefore, our data supports the hypothesis that the paradoxical relationship between adiposity and circulating adiponectin is not dependent upon the accumulation of adipose tissue but rather the concomitant increase in insulin resistance during obesity development [17], [18], [43].

The primary limitation of the present study is that we only studied young males (19–29 yr), which limits the application of our conclusions to other groups. Larger population studies investigating the effects of overfeeding on a wider age distribution which includes females is warranted. In addition, only total circulating adiponectin was measured in this investigation. It has been suggested that high molecular weight adiponectin is the primary physiologically active isoform [44] of adiponectin. However, it has also been shown that low, medium and high molecular weight adiponectin respond to nutritional regulation and exercise the same as total circulating adiponectin [34]. In addition, the influence of inflammatory markers (such as TNF-α, IL-6 and hs-CRP, etc.) on adiponectin regulation is a noteworthy consideration [45]. Therefore, future short-term overfeeding studies investigating influence of inflammatory markers on the regulation and circulation of adiponectin in adipose tissue and peripheral circulation are warranted.

In summary, we investigated the response of circulating adiponectin to a 7-day positive energy challenge mimicking a North American diet in 64 healthy young men. The relationship between adiponectin and adiposity status was also analyzed. The current study revealed that endogenous adiponectin concentration significantly increased within the entire cohort and all three adiposity groups in response to short-term overfeeding. Fasting serum adiponectin levels were found to be similar among normal-weight, overweight and obese young men at baseline and were not associated with adiposity. Negative correlations of adiponectin with %TF, body weight and BMI was observed, but likely arbitrated to insulin resistance. Our data suggests for first time that the increase in adiponectin, in the face of short-term positive energy challenge, may act as a protective mechanism during periods of weight gain against insulin resistance independent of adiposity status and diet composition.
